# Sensory organization of balance control in children with vestibular migraine and recurrent vertigo of childhood

**DOI:** 10.3389/fneur.2022.970610

**Published:** 2022-11-08

**Authors:** Xiaofei Li, Yalan Liu, Yafeng Lyu, Yawei Li, Huirong Jian, Xiaoyi Li, Zhaomin Fan, Haibo Wang, Daogong Zhang

**Affiliations:** Department of Otorhinolaryngology-Head and Neck Surgery, Shandong Provincial ENT Hospital, Shandong University, Jinan, China

**Keywords:** sensory organization test, children, balance, vestibular migraine of childhood, recurrent vertigo of childhood

## Abstract

**Background:**

Migraine plays an important role in some subgroups of children with recurrent vertigo. Moreover, the migraine component varies from definite to possibly absent as defined in this spectrum of three disorders—vestibular migraine of childhood (VMC), probable VMC (pVMC), and recurrent vertigo of childhood (RVC). However, studies on the sensory organization of balance control in these three disorders are rare.

**Objective:**

To explore the balance control of children with RVC, VMC, and pVMC, when the three sensory systems are challenged.

**Method:**

A retrospective analysis was performed on 125 children with VMC (18 female and 15 male; aged 11.64 ± 2.74), pVMC (10 female and eight male; aged 11.78 ± 2.51), and RVC (32 female and 42 male; aged 11.10 ± 2.60). All children in each subtype were divided into groups of children aged ≤ 12 years old and 13–17 years old. Vestibular examination screening and assessment for postural control using the six conditions of the sensory organization test (SOT) were performed. The three primary outcome measures were: equilibrium score (ES), strategy score (SS), and sensory analysis score of the SOT.

**Results:**

Equilibrium score under six different conditions and composite score increased with age (all *P*-values < 0.05). The somatosensory and visual scores also improved with growing (*P*-values < 0.05). However, vestibular scores did not increase significantly with age as the other senses did (*P* > 0.05). In the children ≤ 12 year-old group, children with VMC had a significantly higher visual preference score than those with pVMC and RVC (*P* < 0.05). There was an effect of age on the horizontal HIT. Ocular vestibular evoked myogenic potential (oVEMP), cervical vestibular evoked myogenic potential (cVEMP), and unilateral weakness (UW) values showed no significant difference among three diseases.

**Conclusion:**

Compared with patients at the age of 13–17 years old and with RVC and pVMC (both ≤ 12 years old), children with VMC had a higher degree of reliance on visual signals to maintain their balance and a poorer central integration of peripheral information before reaching 12 years of age. In addition, vision may predominate by weakening vestibular function based on visuo-vestibular interactions. It must be noted that peripheral vestibular examinations could not distinguish the three disease subtypes.

## Introduction

Vertigo or dizziness is not infrequent in pediatric patients. A survey performed among school children revealed that 15% of them have experienced disequilibrium at least once (1). However, specific data on the prevalence of this condition is limited and could be influenced by various factors since children are often incapable of expressing their complaints or describing their symptoms. A retrospective review of 561,151 patients identified a 0.45% prevalence of diagnoses related to balance in children, while another study reported a 5.6% prevalence of dizziness and imbalance in the pediatric population (2, 3), which varied a lot.

Most causes of vertigo and dizziness that occur during childhood and adolescence are benign and treatable. In the past, the most frequent conditions believed to cause vertigo and dizziness during childhood were classified as benign paroxysmal vertigo of childhood (BPVC) and vestibular migraine (VM). However, it is likely that a substantial proportion of pediatric patients with episodic vertigo fit both BPVC and VM criteria. Moreover, published research has shown the likelihood of children with BPVC developing VM later in life (4, 5). Therefore, diagnostic criteria for vestibular migraine of childhood (VMC), probable Vestibular migraine of childhood (pVMC), and recurrent vertigo of childhood (RVC) were established by the Committee for the Classification of Vestibular Disorders of the Barany Society and the Migraine Classification subgroup of the International Headache Society to define subgroups frame more clearly. However, the underlying pathogenesis of these three subgroups, as well as their role in migraine is unclear.

It has been reported that migraine and vertigo in childhood and adolescence has been associated with the presence of behavioral and emotional difficulties (6). However, younger children, especially, are often unable to verbalize “vertigo” in a concrete manner. Therefore, vestibular and balance control assessments are essential for the early identification of vestibular and balance dysfunctions in children. Unlike adults, children's central nervous integration and peripheral sensory systems (vestibular, visual, and somatosensory) undergo changes as they develop. Somatosensory function is nearly mature by the age of 5 years, visual contribution reaches adult levels around ages 11–12 years, and vestibular function continues to mature at least through the age of 15–17 years (7). Strategies for weighing sensory information change as maturation occurs. Meanwhile, age, gender, height, and body mass index (BMI) all need to be accounted for in child vertigo assessment.

In this study, sensory organization test (SOT), postural control, and vestibular tests were explored in children with RVC, VMC, and pVMC to compare the clinical characteristic of these three subgroups.

## Methods

### Participants

**Figure 1:**
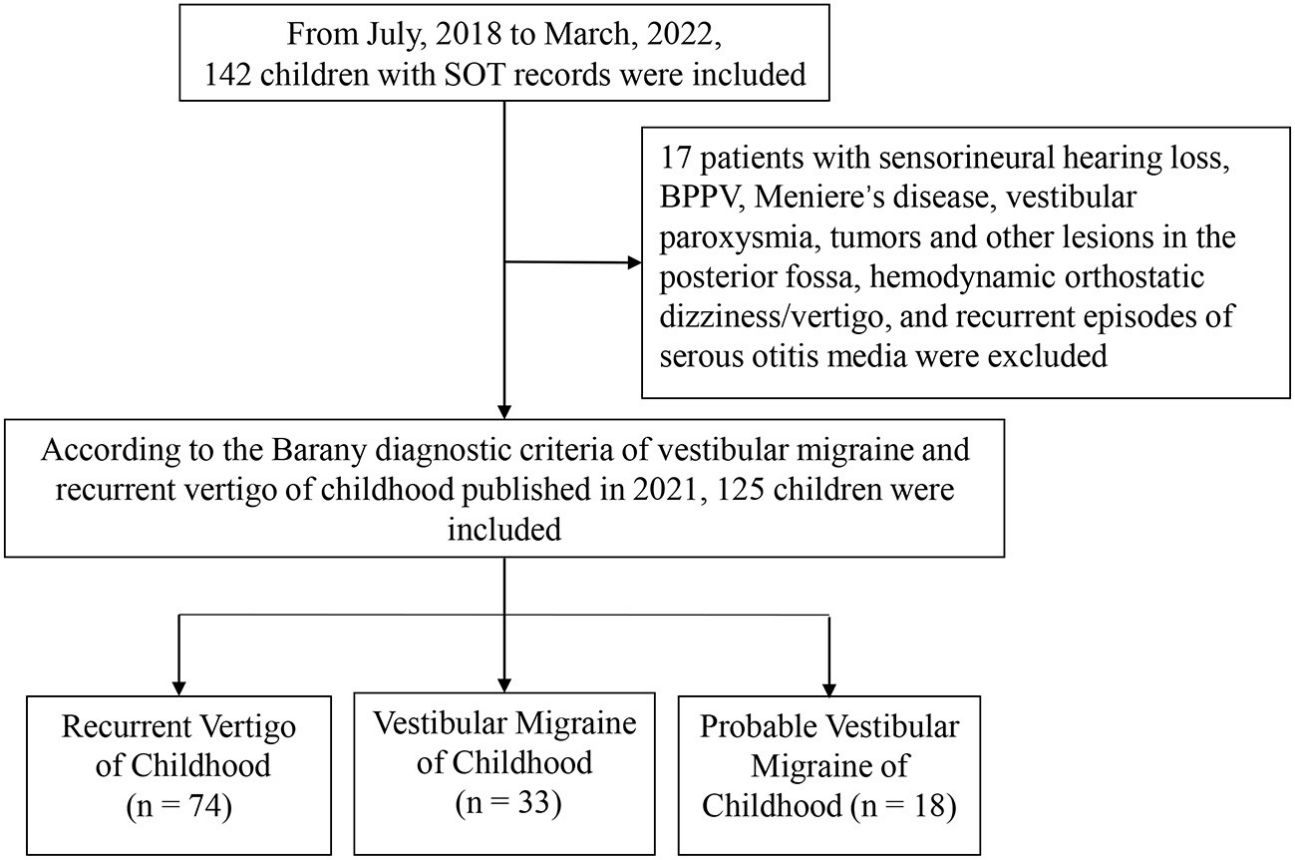
Flow chart.

The medical documents of 142 children who visited our vertigo clinic, from July 2018 to March 2022, with complaints of vertigo/dizziness were retrospectively analyzed (Figure 1). They were diagnosed according to the diagnostic criteria consensus document of the Classification Committee of Vestibular Disorders of the Barany Society and the International Headache Society in 2021 (8). Over-all, 33 cases with vestibular migraine of childhood (aged 11.64 ± 2.74), 18 cases with probable vestibular migraine of childhood (aged 11.78 ± 2.51), and 74 cases with recurrent vertigo of childhood (aged 11.10 ± 2.60) were included. According to the age division of Chinese children, the patients were divided into preschoolers (3–6 years old), early school age (7–12 years old), and adolescents (13–17 years old). The small group of 3–6-year-olds were merged with the group of 7–12-year-olds because they were often uncooperative in some of our tests (mainly SOT and caloric test). Thus, cases in each subtype were divided into groups of ≤ 12 years old and 13–17 years old. Meanwhile, 17 patients who were diagnosed with sensorineural hearing loss, benign paroxysmal positional vertigo, Meniere's disease, tumors and other lesions in the posterior fossa, hemodynamic orthostatic dizziness/vertigo, and recurrent episodes of serous otitis media were excluded.

Medical documents were used with written consent from all patients' guardians. The study was approved by the Shandong Provincial ENT Hospital Ethical Committee.

### Procedures and measures

All the included patients completed vestibular examination screening, including video head impulse test (vHIT), ocular vestibular evoked myogenic potential (oVEMP), cervical vestibular evoked myogenic potential (cVEMP), and caloric tests. They also underwent an assessment for postural control *via* the SOT. The SOT protocol consists of trials under six different sensory conditions: (1) eyes open, surround stable, and platform stable, (2) eyes closed, surround stable, and platform stable, (3) eyes open, sway-referenced surround, platform stable, (4) eyes open, surround stable, sway-referenced platform, (5) eyes closed, surround stable, sway-referenced platform, and (6) eyes open, sway-referenced surround, and sway-referenced platform. By limiting the conditions of the sensory input, SOT forces the individual to reweight another sensory input to maintain postural control. The details of the testing conditions and parameters are summarized in Supplementary Tables 1, 2 (9).

The three primary outcome measures of SOT were: equilibrium score (ES), strategy score (SS), and sensory analysis score of the SOT. Equilibrium score (ES1–ES6) means the stability of center of gravity in the six different challenges. The more stable, the higher the score is. Strategy score reflects that a person chooses to a hip strategy or ankle strategy when facing six different challenges. An ankle strategy means a higher score and a hip strategy means a lower score. The score is calculated mainly depend on the horizontal shear force in anterior-posterior axis. Sensory ratio analysis reflects the ability to use the ratio of visual/vestibular/ somatosensory signal to maintain balance facing six different challenges. The calculation formula can be found in the Supplementary Table 2.

### Data analysis

The mean of the three trials of the six SOT conditions was determined for further analysis. Data are shown as mean and standard deviation. Multivariate analysis of variance were performed to compare the significant difference of variables at different ages (≤ 12 years old and 13–17 years old) and diseases (RVC, VMC, and pVMC). Multiple comparisons were performed followed by the Bonterroni test. A significance level of 0.05 was adopted.

## Results

First, detailed basic characteristics of the study group are shown in Table 1, including the ages, gender, height, weight, and body mass index.

**Table 1:** Demographic details of the patients.

	≤ **12 years**			>**12 years**		
	**RVC** ***n* = 48**	**VMC** ***n* = 19**	**pVMC** ***n* = 10**	**RVC** ***n* = 26**	**VMC** ***n* = 14**	**pVMC** ***n* = 8**
Age	9.60 (1.81)	9.79 (2.00)	10.00 (1.83)	13.88 (1.11)	14.14 (1.01)	14.00 (0.93)
Gender (F:M)	14:34	8:11	6:4	18:8	10:4	4:4
Height (cm)	143.96 (14.14)	145.00 (14.76)	141.29 (19.80)	163.45 (9.44)	162.20 (7.17)	169.55 (9.09)
Weight (kg)	42.17 (19.64)	37.42 (12.87)	37.15 (13.24)	58.62 (17.12)	58.14 (11.82)	66.25 (16.85)
Body mass index (kg/m^2^)	20.27 (9.68)	17.46 (4.50)	18.08 (3.26)	21.88 (5.54)	22.03 (3.85)	22.84 (4.33)

Data are shown as mean (SD).

F, female; M, male.

### Equilibrium score

The data showed a significant effect of age on each variable, with greater scores in the >12 years group. No effect of disease was observed. The equilibrium score under six different conditions and composite score increased with age growing (all *P*-values < 0.05, Tables 2, 3).

**Table 2:** Mean values of variables in different ages and diseases.

	≤ **12 years**			>**12 years**		
	**RVC *n* = 48**	**VMC *n* = 19**	**pVMC *n* = 10**	**RVC *n* = 26**	**VMC *n* = 14**	**pVMC *n* = 8**
**Equilibrium score**						
Condition 1	90.54 (3.65)	91.10 (4.47)	91.67 (2.60)	92.93 (5.23)	93.56 (1.43)	93.73 (2.77)
Condition 2	87.42 (4.77)	86.28 (6.80)	86.48 (4.91)	91.19 (4.02)	91.06 (2.48)	92.73 (2.05)
Condition 3	85.12 (6.37)	87.25 (4.67)	85.07 (4.83)	89.72 (6.14)	89.68 (3.53)	88.98 (4.80)
Condition 4	68.98 (14.69)	65.22 (14.70)	65.70 (19.35)	75.96 (18.66)	81.65 (8.86)	73.10 (31.97)
Condition 5	54.72 (15.77)	45.64 (20.03)	48.25 (19.03)	57.62 (26.70)	66.61 (12.80)	54.17 (29.55)
Condition 6	44.07 (20.92)	45.55 (20.68)	32.70 (23.43)	58.03 (24.56)	61.29 (24.56)	61.29 (20.83)
Composite	66.92 (10.68)	64.84 (11.47)	62.40 (12.19)	73.54 (14.94)	77.50 (7.50)	70.75 (19.97)
**Sensory ratio analysis**						
Somatosensory	96.67 (4.20)	94.89 (4.41)	94.20 (5.61)	98.35 (5.01)	97.50 (2.88)	99.00 (1.93)
Visual	75.67 (14.76)	72.37 (16.05)	71.80 (21.58)	81.15 (18.44)	87.50 (10.07)	77.75 (33.26)
Vestibular	60.19 (16.64)	49.79 (21.61)	53.00 (21.70)	61.04 (28.33)	71.50 (14.06)	57.25 (31.14)
Visual preference	91.02 (14.43)	103.37 (20.99)	87.10 (12.71)	100.38 (17.46)	95.93 (12.52)	98.00 (6.89)
**Strategy score**						
Condition 1	95.73 (1.73)	95.90 (1.35)	96.07 (1.14)	95.22 (3.93)	95.71 (1.21)	95.35 (1.36)
Condition 2	94.57 (2.02)	94.90 (1.93)	94.45 (3.27)	94.54 (2.68)	95.32 (1.18)	94.46 (1.61)
Condition 3	93.98 (3.09)	95.14 (1.41)	93.12 (3.68)	94.15 (3.47)	94.86 (1.96)	92.21 (3.41)
Condition 4	85.06 (5.77)	86.71 (5.49)	85.98 (5.70)	84.28 (9.37)	86.43 (4.97)	87.35 (3.62)
Condition 5	78.62 (9.93)	82.43 (7.67)	79.70 (8.09)	78.02 (9.43)	79.05 (7.49)	77.33 (9.85)
Condition 6	80.91 (7.61)	84.41 (6.33)	80.62 (9.33)	82.19 (5.80)	80.67 (7.40)	75.54 (9.66)
**Peripheral vestibular tests**						
HIT RA	1.01 (0.13), *n* = 41	1.03 (0.07), *n* = 17	1.05 (0.07), *n* = 10	0.99 (0.10), *n* = 25	1.07 (0.08), *n* = 14	1.01 (0.11), *n* = 7
HIT RH	0.99 (0.08), *n* = 41	1.02 (0.07), *n* = 17	1.02 (0.03), *n* = 10	0.98 (0.09), *n* = 25	0.96 (0.11), *n* = 14	0.93 (0.13), *n* = 7
HIT RP	1.00 (0.07), *n* = 41	0.97 (0.07), *n* = 17	0.99 (0.10), *n* = 10	0.95 (0.08), *n* = 25	0.97 (0.10), *n* = 14	0.96 (0.10), *n* = 7
HIT LA	1.02 (0.13), *n* = 41	1.04 (0.08), *n* = 17	1.00 (0.09), *n* = 10	0.97 (0.14), *n* = 25	1.06 (0.09), *n* = 14	0.98 (0.05), *n* = 7
HIT LH	0.98 (0.11), *n* = 41	0.91 (0.25), *n* = 17	1.02 (0.06), *n* = 10	0.99 (0.07), *n* = 25	0.95 (0.11), *n* = 14	0.92 (0.18), *n* = 7
HIT LP	0.98 (0.06), *n* = 41	0.96 (0.12), *n* = 17	1.00 (0.09), *n* = 10	0.97 (0.08), *n* = 25	0.98 (0.09), *n* = 14	0.91 (0.11), *n* = 7
UW (%)	25.47 (21.47), *n* = 41	26.16 (16.57), *n* = 15	*n* = 0	23.82 (21.37), *n* = 23	17.90 (19.14), *n* = 14	14.38 (17.94), *n* = 8
cVEMP asymmetry ratio of amplitude (%)	29.69 (31.98), *n* = 42	31.19 (31.54), *n* = 18	*n* = 0	34.21 (37.65), *n* = 24	33.00 (37.58), *n* = 14	27.41 (33.71), *n* = 7
oVEMP asymmetry ratio of amplitude (%)	30.50 (34.50), *n* = 42	38.42 (33.48), *n* = 17	*n* = 0	47.25 (43.79), *n* = 21	40.15 (40.38), *n* = 14	41.21 (45.66), *n* = 6

Data are shown as mean (SD).

HIT, video head impulse test; L, left; R, right; A, anterior; H, horizontal; P, posterior; UW, unilateral weakness; cVEMP, cervical vestibular evoked myogenic potential; oVEMP, ocular vestibular evoked myogenic potential.

Note, not all the children completed all the peripheral vestibular tests.

**Table 3:** Tests of between-subject effects of variables.

	**Age**				**Disease**					Age*disease	
	** ≤ 12 years**	**>12 years**	***F***	***P*-values**	**RVC**	**VMC**	**pVMC**	***F***	***P*-values**	***F***	***P*-values**
**Equilibrium score**											
Condition 1	91.10 (0.54)	93.41 (0.64)	7.719	0.006	91.73 (0.48)	92.32 (0.69)	92.70 (0.93)	0.546	0.581	0.017	0.983
Condition 2	86.73 (0.65)	91.66 (0.76)	24.231	0.000	89.31 (0.57)	88.67 (0.83)	89.61 (1.12)	0.286	0.751	0.524	0.594
Condition 3	85.81 (0.78)	89.46 (0.91)	9.246	0.003	87.42 (0.69)	88.46 (0.99)	87.02 (1.34)	0.502	0.606	0.404	0.669
Condition 4	66.63 (2.36)	76.91 (2.74)	8.077	0.005	72.47 (2.07)	73.44 (2.99)	69.40 (4.03)	0.335	0.716	0.892	0.413
Condition 5	49.54 (2.79)	59.47 (3.25)	5.365	0.022	56.17 (2.45)	56.12 (3.54)	51.21 (4.77)	0.456	0.635	2.233	0.112
Condition 6	40.77 (3.07)	57.89 (3.57)	13.189	0.000	51.05 (2.69)	53.42 (3.90)	43.53 (5.25)	1.184	0.310	0.213	0.808
Composite	64.72 (1.72)	73.93 (2.00)	12.245	0.001	70.22 (1.50)	71.17 (2.18)	66.58 (2.93)	0.838	0.435	0.651	0.523
**Sensory ratio analysis**											
Somatosensory	95.25 (0.60)	98.28 (0.70)	10.817	0.001	97.51 (0.53)	96.20 (0.76)	96.60 (1.03)	1.095	0.338	0.928	0.398
Visual	73.28 (0.44)	82.14 (2.83)	5.617	0.019	78.41 (2.14)	79.93 (3.09)	74.78 (4.16)	0.500	0.608	0.867	0.423
Vestibular	54.33 (2.99)	63.26 (3.48)	3.803	0.054	60.61 (2.62)	60.65 (3.79)	55.13 (5.10)	0.493	0.612	2.609	0.078
Visual preference	93.83 (2.17)	98.10 (2.53)	1.643	0.202	95.70 (1.91)	99.65 (2.76)	92.55 (3.71)	1.297	0.277	3.516	0.033
**Strategy score**											
Condition 1	95.90 (0.31)	95.43 (0.36)	0.969	0.327	95.48 (0.27)	95.81 (0.40)	95.71 (0.53)	0.264	0.769	0.091	0.913
Condition 2	94.64 (0.30)	94.77 (0.35)	0.078	0.780	94.56 (0.27)	95.11 (0.39)	94.45 (0.52)	0.829	0.439	0.120	0.887
Condition 3	94.08 (0.41)	93.74 (0.48)	0.290	0.591	94.07 (0.36)	95.00 (0.52)	92.66 (0.70)	3.596	0.030	0.254	0.776
Condition 4	85.92 (0.90)	86.02 (1.05)	0.006	0.940	84.67 (0.79)	86.57 (1.14)	86.67 (1.54)	1.278	0.282	0.193	0.825
Condition 5	80.25 (1.27)	78.13 (1.47)	1.185	0.279	78.32 (1.11)	80.74 (1.61)	78.52 (2.16)	0.798	0.453	0.271	0.763
Condition 6	81.98 (1.02)	79.47 (1.19)	2.579	0.111	81.55 (0.90)	82.54 (1.30)	78.08 (1.74)	2.201	0.115	2.048	0.134
**Peripheral vestibular tests**											
HIT RA	1.03 (0.02)	1.02 (0.02)	0.241	0.625	1.00 (0.01)	1.05 (0.02)	1.03 (0.03)	2.285	0.107	0.955	0.388
HIT RH	1.01 (0.01)	0.95 (0.02)	8.370	0.005	0.99 (0.01)	0.99 (0.02)	0.97 (0.02)	0.193	0.825	1.506	0.227
HIT RP	0.99 (0.01)	0.96 (0.01)	2.093	0.151	0.97 (0.01)	0.97 (0.01)	0.97 (0.02)	0.000	1.000	1.051	0.353
HIT LA	1.03 (0.02)	1.01 (0.02)	0.596	0.442	1.00 (0.01)	1.05 (0.02)	1.00 (0.03)	2.522	0.085	0.986	0.376
HIT LH	0.97 (0.02)	0.95 (0.02)	0.422	0.517	0.98 (0.02)	0.93 (0.02)	0.97 (0.03)	1.759	0.177	1.536	0.220
HIT LP	0.98 (0.01)	0.95 (0.02)	2.419	0.123	0.97 (0.01)	0.97 (0.02)	0.95 (0.02)	0.390	0.678	2.235	0.112
UW (%)	25.82 (3.06)	18.70 (3.31)	1.164	0.283	24.65 (2.64)	22.03 (3.76)	14.74 (7.16)	0.536	0.587	0.518	0.473
cVEMP asymmetry ratio of amplitude (%)	30.44 (4.81)	31.54 (5.76)	0.178	0.674	31.95 (4.37)	32.09 (6.09)	27.42 (12.91)	0.096	0.909	0.033	0.857
oVEMP asymmetry ratio of amplitude (%)	34.46 (5.45)	42.87 (6.76)	1.177	0.281	38.88 (5.07)	39.28 (6.85)	41.22 (15.49)	0.012	0.988	0.777	0.380

Data are shown as mean (SD).

HIT, video head impulse test; L, left; R, right; A, anterior; H, horizontal; P, posterior; UW, unilateral weakness; cVEMP, cervical vestibular evoked myogenic potential; oVEMP, ocular vestibular evoked myogenic potential.

Note, not all the children completed all the peripheral vestibular tests.

### Somatosensory, visual and vestibular score

**Figure 2:**
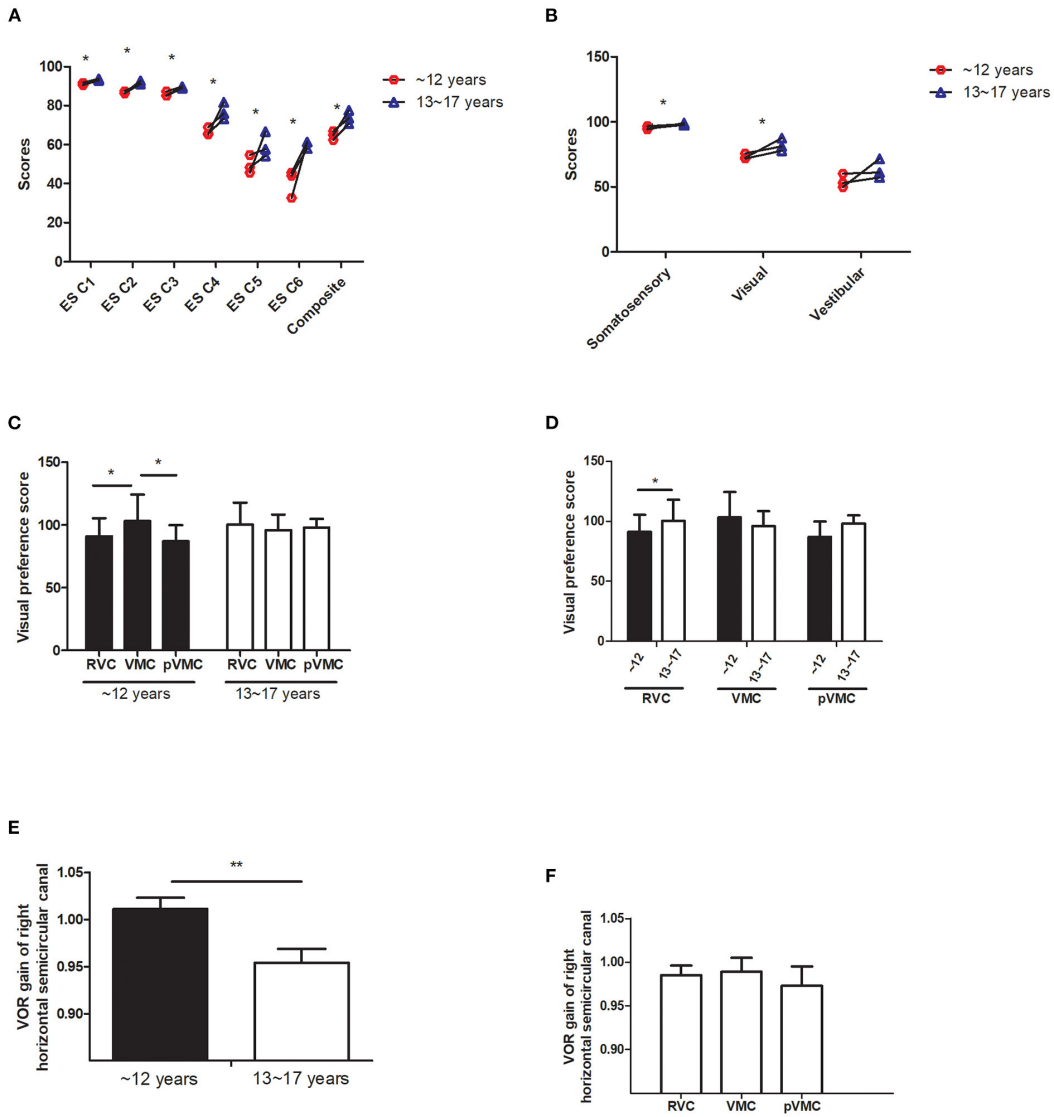
Sensory organization test analysis. **(A)** Equilibrium score. **(B)** Sensory ratio analysis. **(C,D)** Visual preference score in different age groups and diseases. **(E,F)** VOR gain value of horizontal semicircular canal in different age groups and disease.ES C, Equilibrium score condition; SS C, strategy score condition; VMC, vestibular Migraine of Childhood; pVMC, probable Vestibular Migraine of Childhood; RVC, Recurrent Vertigo of Childhood; VOR, vestibular ocular reflex. *P < 0.05, **P < 0.05.

A significant effect of age on somatosensory and visual score, with greater scores in the >12 years group; no effect of disease, indicating that somatosensory and visual scores improved with growing (*P* = 0.001, *P* = 0.019). However, vestibular scores did not increase significantly with age as the other senses did (*P* > 0.05; Table 3, Figures 2A,B).

### Visual preference

Visual preference score means the degree to the subject who relies on visual signal to maintain balance (correct/incorrect information). The visual preferece score analysis indicated no effect of age (*F* = 1.643, *P* = 0.202), no effect of disease (*F* = 1.297, *P* = 0.277), a significant effect of age^*^disease interaction, with a significant greater score in the VMC disease group, compared with either tje RVC or the pVMC group (*P* = 0.013, *P* = 0.027, respectively), only in the ≤ 12 years group. There are significant differences between the two age groups in RVC (*P* = 0.015). No significant difference was found between different ages in children with VMC or pVMC (*P* > 0.05; Tables 3, 4, Figures 2C,D).

**Table 4:** Pairwise comparison of visual preference score.

**Comparison**	**Visual preference score**	***P*-values**
≤ 12 years: RVC vs. VMC	91.02 (14.43) vs. 103.37 (20.99)	0.013
≤ 12 years: RVC vs. pVMC	91.02 (14.43) vs. 87.10 (12.71)	1.000
≤ 12 years: VMC vs. pVMC	103.37 (20.99) vs. 87.10 (12.71)	0.027
>12 years: RVC vs. VMC	100.38 (17.46) vs. 95.93 (12.52)	1.000
>12 years: RVC vs. pVMC	100.38 (17.46) vs. 98.00 (6.89)	1.000
>12 years: VMC vs. pVMC	95.93 (12.52) vs. 98.00 (6.89)	1.000
RVC: ≤ 12 years vs. >12 years	91.02 (14.43) vs. 100.38 (17.46)	0.015
VMC: ≤ 12 years vs. >12 years	103.37 (20.99) vs. 95.93 (12.52)	0.180
pVMC: ≤ 12 years vs. >12 years	87.10 (12.71) vs. 98.00 (6.89)	0.145

Data were shown as mean (SD). Bonterroni test was performed for pairwise comparison. P value here are shown as N* 0.05. N means the number of tests.

VMC, vestibular Migraine of Childhood; pVMC, probable Vestibular Migraine of Childhood; RVC, Recurrent Vertigo of Childhood.

### Strategy score

There was no significant effect of age on each variable; no effect of disease with the exception of condition 3, whose score was significantly lower in the pVMC group compared with VMC group (*P* = 0.030) in both age groups.

### Peripheral vestibular tests

Video head impulse test reflect the high frequency function of each semicircular canal. The main outcome parameter was vHIT VOR gain by evaluating the relation between eye and head velocity. The data revealed an effect of age on the horizontal HIT, with a greater VOR gain of right horizantal semecircular canal in the ≤ 12 years group (*F* = 8.370, *P* = 0.005); No effect of disease and age^*^disease (*P* > 0.05; Table 3, Figures 2E,F). The other peripheral vestibular test results, including, unilateral weakness (UW) of caloric test, and asymmetry rate of VMEP were compared and had no significant differences (all *P*-value > 0.05). Note that not all the patients compete the peripheral vestibular tests as indicated in Table 2.

Last, some symptomatological data were complemented on when the test were done, age of onset age, attack duration and number of attack in the last 3 months. Almost all the patients have had attacks within about 1 week before visit, suggesting that the examination data comes from an active phase (Table 5).

**Table 5:** Symptomatological information.

**Age of onset (years)**		**Interval since the last attack (days)**		**Number of attack in the last 3 months**		**Attack duration (hours)**	
**RVC**	**VMC + pVMC**	**RVC**	**VMC + pVMC**	**RVC**	**VMC + pVMC**	**RVC**	**VMC + pVMC**
9.92 (2.82)	9.84 (3.47)	7.10 (10.23)	6.49 (9.90)	14.48 (23.23)	24.61 (53.74)	8.62 (15.19)	7.54 (21.82)

Data are shown as mean (SD).

VMC, vestibular migraine of childhood; pVMC, probable vestibular migraine of childhood; RVC, recurrent vertigo of childhood.

## Discussion

This study aimed to explore the postural control of children with RVC, VMC, and pVMC, when the three sensory systems are challenged, using computerized dynamic posturography testing. Since differences in postural control and sensory weighting may be attributed to not only neural integration but also anthropometric characteristics (height, weight, BMI etc.) (7), the included patients were divided into different groups depending on their ages. Thus, the current study explore the postural control of children from two point of view, ages and diseases.

As the sensory system and central system develop, the equilibrium ability and pattern of children gradually mature. Significant age-associated increases in overall performance on the SOT were found in healthy children (7). In the current study, the equilibrium score revealed that the ability to use the vestibular input does not increase significantly with age unlike vision and somatosensory, which may be one of the causes of vertigo in children.

The ability to utilize specific sensory inputs effectively develops at different ages. Somatosensory function is nearly mature by the age of 5 years, visual contribution reaches adult levels around ages 11–12 years, and vestibular function continues to mature at least through the age of 15–17 years (7). In the current study, vision dependence difference among three subtype groups was only observed in children aged ≤ 12 years rather than in older children, which might be because visual signals are dominant before 12 years of age. Moreover, young children are more dependent on visual cues, although the visual system is less mature than other sensory inputs for postural control (10). No differences in the visual preference ratio were observed among three subtype groups in older children (13–17 years old), indicating that the factors of age and development should be taken into consideration in the diagnosis and intervention of the three subtypes.

Furthermore, children with VMC showed a higher visual preference score, suggesting that those with VMC were more dependent on visual signals (correct/incorrect information) to maintain balance than those with pVMC and RVC at the same age (≤ 12 years old). Previous study reported that the cerebellum nodulus and uvula (integration centers for canal and otolith signals) have been found to have increased sensitivity in vestibular migraine patients (11). This increased sensitivity could cause increased inhibition of the vestibular nuclei, as well as inhibition of the velocity storage of vestibular signals, which would lead to increased dependence on visual signals. It is postulate that VM patients have impaired visuo-vestibular cortical interactions, which in turn disrupts normal vestibular function (12). Therefore, it can be inferred that the peripheral vestibular signals of those with VMC are impaired induced by increased inhibition of the vestibular nuclei. In addition, a visual preference suggests that vision may predominate by weakening vestibular function based on visuo-vestibular interactions. We speculated that both mechanisms are involved in the different performances of the three disease subtypes. However, further research is needed to confirm this.

In addition, our data found there is a meaningful difference of visual preference between the two age groups in RVC; there is no significant difference between the two age groups in VMC and pVMC. It is speculated that vertigo disorders in children with or without migraine may be substantially different. Morever, in the case of RVC, the implication is that the timing of the intervention may affect the patient's sensory processing pattern.

Other studies have reported clinical implication of peripheral vestibular impairment in vestibular migraine patients. Woo Seok Kang et al. revealed that abnormal video head impulse and caloric tests in VM patients predicted prolonged preventive medication requirement, suggesting that peripheral vestibular abnormalities are closely related to the development of vertigo in VM patients (13). In our study, vHIT of right horizontal canal had significant difference in ages but not in diseases. It has been reported that healthy population have a higher VOR gain in right side than that of left. Meanwhile, VOR gain value of vHIT decreases with age increasing (14). Therefore, in current study, it is believed that the significant difference of vHIT is mainly attributed to ages and sides, not due to diseases. Alternatively, peripheral vestibular function was not different among these diseases, implying that the difference mainly comes from the central integration and processing of peripheral information rather than the peripheral sensory input. A previous study utilized the functional head impulse test (fHIT) with and without an optokinetic stimulus to unveil a functional vestibular impairment in adult patients with VM, mainly impairing the capability to integrate different vestibular stimuli (15). A similar impairment was also reported for the integration of rotational and gravitational cues (16), as well as visual motion stimulation that disturbed the postural stability of adult patients with VM (17). Some additional studies on pediatric VM revealed that abnormalities are more common on balance tests than on vestibular tests in pediatric VM (18, 19). These studies are consistent with our findings. Therefore, we speculate that for children with vertigo disease, more attention should be paid to the overall balance ability rather than just examining the peripheral vestibular function.

## Limitations

First, this was a cross-sectional study that cannot observe the longitudinal outcomes of the three disease subtypes in the same cases. The current comparison between the two groups of younger and older children also provides some useful information. Second, there was no healthy patients included as control. Thus, the results (no difference) observed in some parameters can only be applied for those with the three disease subtypes, and not for normal patients.

## Conclusion

Compared with patients at the age of 13–17 years old and with RVC and pVMC (both ≤ 12 years old), children with VMC had a higher degree of reliance on visual signals to maintain their balance and a poorer central integration of peripheral information before reaching 12 years of age. In addition, vision may predominate by weakening vestibular function based on visuo-vestibular interactions. It must be noted that peripheral vestibular examinations could not distinguish the three disease subtypes.

## Data availability statement

The original contributions presented in the study are included in the article/Supplementary material, further inquiries can be directed to the corresponding authors.

## Ethics statement

The studies involving human participants were reviewed and approved by Shandong Provincial ENT Hospital Ethical Committee. Written informed consent to participate in this study was provided by the participants' legal guardian/next of kin.

## Author contributions

HW and DZ contributed to the conception of the work. DZ, ZF, and YLyu contributed to the experimental design. YLi, HJ, and XL collected data. XL performed the analysis. XL and YLiu wrote the manuscript. DZ revised the manuscript. All authors contributed to the article and approved the submitted version.

## Funding

This study was funded by Shandong Provincial Natural Science Foundation (No. ZR2020MH179), Taishan Scholars Program of Shandong Province (No. ts20130913), and National Natural Science Foundation of China (No. 81900940).

## Conflict of interest

The authors declare that the research was conducted in the absence of any commercial or financial relationships that could be construed as a potential conflict of interest.

## Publisher's note

All claims expressed in this article are solely those of the authors and do not necessarily represent those of their affiliated organizations, or those of the publisher, the editors and the reviewers. Any product that may be evaluated in this article, or claim that may be made by its manufacturer, is not guaranteed or endorsed by the publisher.
