# Supplementary material for: Sensory organization of balance control in children with vestibular migraine and recurrent vertigo of childhood
Source: Front Neurol. 2022 Nov 8;13:970610. doi: 10.3389/fneur.2022.970610 (PMC9678931; doi:10.3389/fneur.2022.970610)
Supplement: Supplementary file 1 [file Table_1.DOCX]

Supplementary Material

# Supplementary Tables

Supplemental table 1. Testing conditions of the sensory organization test

| Condition | Description | Sensory signals input |
| --- | --- | --- |
| 1 | Eyes open, fixed platform | Somatosensory, visual, vestibular |
| 2 | Eyes closed, fixed platform | Somatosensory, vestibular |
| 3 | Sway-referenced vision, fixed platform | Disturbed vision, somatosensory, vestibular |
| 4 | Eyes open, sway-referenced platform | visual, vestibular |
| 5 | Eyes closed, sway-referenced platform | vestibular |
| 6 | Sway-referenced vision, sway-referenced platform | Disturbed vision, vestibular |
