# Supplementary material for: Sensory organization of balance control in children with vestibular migraine and recurrent vertigo of childhood
Source: Front Neurol. 2022 Nov 8;13:970610. doi: 10.3389/fneur.2022.970610 (PMC9678931; doi:10.3389/fneur.2022.970610)
Supplement: Supplementary file 2 [file Table_2.DOCX]

Supplementary Material

Supplemental table 2. The significance of sensory organization test measurement parameters

| Parameters | Equation | Significance |
| --- | --- | --- |
| Equilibrium score(ES1-ES6) | =12.5°-[(θmax-θmin)/12.5°]x100* | 100% means perfect stability, 0% means falling |
| Composite score | = [ES1 + ES2 + 3(ES3 + ES4 + ES5 + ES6)] / 14 | Global determination of normal versus abnormal performance |
| Somatosensory ratio | = ES2 / ES1 | The ability to use somatosensory signal to maintain balance |
| Visual ratio | = ES4 / ES1 | The ability to use visual signal to maintain balance |
| Vestibular ratio | = ES5 / ES1 | The ability to use vestibular signal to maintain balance |
| Visual preference ratio | = ES3 + ES6 / ES2 + ES5 | Degree to the subject relies on visual signal to maintain balance(correct/incorrect information) |
| Strategy score | = [ 1 - (SHmax - SHmin) / 25] x 100** | 100% means a predominance of an ankle strategy to maintain equilibrium, 0% means predominantly a hip strategy |

*θmax is the largest center of gravity sway angle in the anterior-posterior axis attained by the participant and θmin is the smallest.

** SHmax is the greatest horizontal shear force in anterior-posterior axis observed and SHmin is the lowest.
